# Treatment goals for adults with early treated PKU should be determined by evidence-based shared decision making between patients and their medical team

**DOI:** 10.1186/s13023-026-04376-5

**Published:** 2026-06-02

**Authors:** Mirjam Langeveld, Sandra Sirrs, Robin Lachman, Timothy Fazio, Reena Sharma, Athanasia Ziagaki, Peter Reismann, Michel Tchan, Christel Tran, Melanie M. van der Klauw, Daphne Vassiliou, Elaine Murphy

**Affiliations:** 1 Department of Endocrinology and Metabolism, Amsterdam UMC Location University of Amsterdam and Amsterdam Gastroenterology, Endocrinology and Metabolism Research Institute, Amsterdam, The Netherlands; 2 Vancouver General Hospital, Vancouver, Canada; 3 Charles Dent Metabolic Unit, National Hospital for Neurology and Neurosurgery, London, UK; 4 Metabolic Diseases Unit, Royal Melbourne Hospital, Parkville, VIC Australia; 5 Department of Medicine, Melbourne Medical School, University of Melbourne, Parkville, VIC Australia; 6 Adult Inherited Metabolic Diseases, Salford Care Organisation, Northern Care Alliance NHS Foundation Trust, Salford, UK; 7 Department of Endocrinology and Metabolic Diseases, Charité - Universitätsmedizin Berlin, Berlin, Germany; 8 Department of Internal Medicine and Oncology, Semmelweis University, Budapest, Hungary; 9 Department of Genetic Medicine, Westmead Hospital, Westmead, NSW 2145 Australia; 10 Sydney Medical School, University of Sydney, Camperdown, NSW 2050 Australia; 11 Division of Genetic Medicine, University of Lausanne and University Hospital of Lausanne, Lausanne, Switzerland; 12 Department of Endocrinology, University of Groningen, University Medical Center Groningen, Groningen, The Netherlands; 13 Department of Endocrinology, Karolinska University Hospital and Department of Molecular Medicine and Surgery, Stockholm, Sweden

**Keywords:** Phenylketonuria, PKU, Adult, Treatment target, Cognitive functioning

## Abstract

This opinion article focusses on treatment targets in adults with early treated PKU (etPKU), specifically the recommended phenylalanine (Phe) level. The current body of evidence on the relationship between Phe levels at different times in life and neurocognitive functioning and quality of life in adults with etPKU is summarized and discussed. Based on this, recommendations on metabolic control throughout adult life are made. Maintaining plasma Phe < 600 umol/L up to (at least) age 20 years may be advisable. There is however a lack of robust evidence to support a specific single phenylalanine target level that is applicable to all adults and, in later adulthood, a patient-centred approach to optimize nutritional status and achieve individualised treatment goals should be taken. The available evidence indicates reduced processing speed and attentional difficulties with higher Phe concentrations in only a proportion of adult patients. These are potentially reversible with lowering of Phe levels. Patients may wish to achieve lower phenylalanine levels because they experience better mood or cognitive functioning, and the levels where they perceive these benefits are variable (e.g. <600, < 800 or < 1000 umol/L). Equally, patients may wish to avoid potential, as yet undiscovered, long term negative consequences of higher levels. All patients wishing to lower their Phe levels should receive full support to reach their goals. In some cases, subjective symptoms are the reason patients want to reduce their Phe levels. In such cases, subjective symptoms should be clearly defined and quantified, and the relationship between their occurrence and phenylalanine level, as well as improvement with lower phenylalanine levels, should be clearly established before treatment is intensified. Patients who, after having received and understood all currently available evidence on outcomes, do not wish to lower their Phe levels, should not be pressed to do so. In order to further understand the correlation between Phe levels at the different stages of life and neurological and cognitive functioning of etPKU patients a large, independent, multicentre registry needs to be established. This is urgently needed to facilitate rational treatment of PKU in adulthood, preventing both over- and undertreatment and facilitating cost-effective use of new and currently available drugs.

**Supplementary Information:**

The online version contains supplementary material available at 10.1186/s13023-026-04376-5.

## Background

### Methodological aspects

This opinion article is written in response to the latest American [1] and European [2] guidelines on treatment of phenylketonuria (PKU). It is not a full structured review or guideline but and opinion article focussed on the evidence underlying the adult treatment goals for people whose PKU was identified by newborn screening and whose treatment was started in the newborn period (i.e. early treated PKU (etPKU)). In reaching this opinion, we considered all the relevant articles cited by either guideline. In addition, we also looked at articles identified by subtopic specific searches (e.g. cognitive functioning or wellbeing), with search strategies based on those used in the revised European PKU guideline, (see supplemental material 1 of Wegberg et al. [2] and those published after the closure of the American and European guideline searches were included. This article summarizes the current evidence on which recommendations can be based, and focusses on quality aspects of the studies such as sample size, description of characteristics of the studies’ participants, study design, statistical analysis and year of publication. The last is important, since newer studies will better reflect the impact of more recent treatment options compared to older studies (e.g. studies on the cognitive profiles of young adults published in the 1990s reflect the effect of care in the 1970s and 1980s, with less palatable protein substitutes and a high likelihood of treatment being discontinued in early adolescence in many countries). This paper does not deal with treatment targets for women with etPKU who are pregnant or planning pregnancy since treatment targets in pregnancy are derived from rigorous treatment trials which confirm the benefits of tight Phe control to avoid damage to the unborn child.

### Phenylketonuria

Phenylketonuria (#261600) is an autosomal recessive inherited disorder of protein metabolism caused by deficiency of the enzyme phenylalanine hydroxylase. Without treatment it results in elevated blood phenylalanine concentrations and causes intellectual disability. Since the introduction of newborn screening (NBS) and early dietary treatment, individuals with PKU no longer develop profound and irreversible intellectual disability. In children, it is very clear that maintaining target Phe levels prevents loss of IQ, with a rise of 400 umol/L in Phe results in a loss of 0.5 SD IQ [3, 4]. If adequate treatment is started early and maintained throughout childhood, adults with PKU live independent lives with educational, occupational and social outcomes comparable to their unaffected peers [5]. Some adults with etPKU have discontinued PKU diet in adulthood, but nonetheless remain well with normal neurocognitive profiles [5]. Nonetheless, it is true that, despite wide inter-individual variability, as a group, adults with etPKU do have impairments on neurocognitive testing [6]. These impairments may have developed at any period in the patient’s life up to the time of testing. The neurocognitive profile is very different from that seen in patients with late-diagnosed PKU. We propose calling the neuropsychological and neurological sings and symptoms related to PKU present in etPKU patients in adulthood “residual PKU”.

Defining the features of residual PKU is not straightforward. Determining whether an individual patient is presenting with signs or symptoms which are due to residual PKU can be challenging when complaints such as impaired attention, brain fog, low mood or irritability are issues which are commonly reported in the general population as well: they may have many causes other than PKU and we need to establish if these problems are more common in etPKU patients than in the general population. For patients with the neurocognitive profile of residual PKU, we need longitudinal follow up to determine whether this is fixed or progressive. We also need to determine whether the features of residual PKU are related to current Phe concentrations in adulthood, or to historic metabolic control in childhood or to a cumulative effect of both. Finally, before setting a target Phe level for adults with etPKU, we need to be clear whether any elements of residual PKU can be reversed or prevented by improving metabolic control and reducing Phe levels.

It is important to define the actual benefit of Phe lowering treatment in adulthood to sure that currently available and soon to be launched orphan drugs for the treatment of PKU are prescribed in a cost-effective manner. This includes identifying which adult etPKU patient groups benefit from Phe lowering therapy (all or specific age groups?). If the benefit is a change in neuropsychological test scores, it needs to be established whether or not this is reflected in better psychosocial functioning in daily life and what the magnitude of these effects are. This can then be weighed against the physical, psychological and financial burden of the treatment. We believe that determining which patients will benefit from therapy is a key task for physicians and dieticians treating adults. It is inappropriate from a patient perspective to expose a patient to the risks of side effects with new therapies if they will not benefit. Giving expensive therapies to patients who will not benefit is also deleterious from a resource perspective and such considerations are increasingly important as healthcare budgets worldwide become stretched with the mounting costs of new treatments and an aging population.

### Questions addressed in this paper

In this paper we try to answer the following questions in order to be able to advise adults on whether phenylalanine lowering for life is recommended:

1. What is the cognitive profile of early treated adults with PKU and how does this relate to concurrent and historical Phe levels?
2. What is the current quality of life of early treated adults with PKU (including mood, educational, occupational and social functioning) and how does this relate to concurrent and historical Phe levels?
3. Is there evidence for new onset progressive neurological or cognitive impairment in early treated adults who discontinue or relax phenylalanine lowering treatment in adulthood?
4. Do interventions to alter Phe concentrations in adulthood change cognitive, occupational and social functioning?
5. Should there be a recommended Phe concentration for all non-pregnant adults? Or should it be different for different (age) subgroups?

## Summary of findings from the literature

### Cognitive functioning of early treated adults

A meta-analysis by Romani et al. included defined data from articles (published up to May 2022) reporting cognitive performance in early treated adults with PKU [6]. The goal was to report effect sizes for impairment across different cognitive functions. Effect size in this case means the difference between the etPKU patients and healthy control subjects expressed as standard deviation from the mean. Individuals were included if they were (1) treated before the age of 3 months and (2) were 16 years and older at the time of assessment. Comparisons were made to an age matched control group. IQ was on average 0.6 SD lower in individuals with PKU compared to controls. Scores significantly below that of the control groups were found in the following domains (in order of effect size from highest (-1.02 SD) to lowest (-0.18 SD)): reasoning, visuo-spatial attention reaction time (RT), sustained attention, visuo-motor control, flexibility and planning, visual learning/long term memory, social cognition, higher language skills, verbal short-term memory/working memory and visuo-spatial skills. For all effects there was a wide confidence interval within studies and significant variation between studies. There was a positive relationship between average Phe at time of testing and the effect sizes, with worse performance in patients with higher Phe concentrations. Phe concentrations at the time of testing were related to the age of treatment initiation (higher levels in those who started treatment later) and year of publication of the study (higher levels in older studies). Historical (neonatal, childhood, adolescence) Phe concentrations were generally not available and so were not accounted for in this meta-analysis. A more recent French study in 187 etPKU patients lacked information on age (days) at start and discontinuation of treatment [7]. Thus no conclusions can be drawn on whether the relationship between higher Phe levels at the time of testing and worse cognitive performance was a result of higher childhood Phe levels, higher Phe levels in adulthood or a combination of both. Another recent German study in 36 etPKU patients analysed the results of executive function tests in relation to metabolic control at different stages in life. However, since the relationship between current and recent Phe and the test results was not corrected for historical Phe levels, no conclusion can be drawn regarding their relative influence [8].

Phe concentrations in the neonatal period, childhood and adolescence have a clear negative correlation with cognitive functioning in adulthood [9–13]. Three studies correct for childhood and adolescent Phe control when assessing the effect of Phe levels at the time of cognitive testing in adulthood (results summarized in Table 1). In the largest study by Aitkenhead et al.154 early treated adults (start of treatment within the first month of life) underwent cognitive testing and were compared to population norms. Significantly lower z-scores (-0.33 to -0.61) for adults with PKU versus population norms were found for attention, working memory, executive function, and processing speed. The percentage of adults with PKU with z-scores below 1.5 SD of normative data for individual tests ranged from 7 to 23%, with 53% of participants scoring normally on all tests. No significant differences were found for learning and memory. Blood Phe levels on the day of testing showed a negative relationship with processing speed and learning scores, but only the relationship for processing speed remained significant after adding historical Phe levels to the model [5].

**Table 1:** The relation between childhood/adolescence and current/recent Phe levels on cognitive functioning in adult individuals with early treated PKU

First author Year	Number of patients	Type of patients	Age at assessment (years)	Cognitive domains assessed	Periods for which Phe levels are analysed	Significant relations between Phe levels and cognitive functioning	Significant relations between current/recent Phe levels and cognitive functioning after correction for historical Phe levels	Proportion of patients with normal cognitive testing outcomes	Funding by pharmaceutical or nutritional company of study or employment of author(s)
Aitkenhead 2021 [5]	154	Early-treated adult patients. None treated regularly with BH4.	Mean 32.38 SD 9.04	Attention, learning, working memory, language, executive function, processing speed	Lifelong. Six-month median results averaged across developmental epochs (0–6 years, 6–12 years, 12–18 years, > 18 years)	Higher Phe levels correlated with slower processing speed and poorer learning scores.	Only processing speed showed a significant correlation once corrected for historical Phe, with an increase of 1000umol/L associated with a processing speed of -1.05SD.	A range of 97.6% to 76.51% depending on domain assessed (at or above − 1.5SD below the mean; expected rate 93.32%). 53% normal in all domains.	Part-funded through Nutricia Metabolic Research Fund.
Jahja 2017 [14]	57	Early-treated adult patients, including 12 treated with BH4.	Mean 27.7 SD 6.0	IQ, working memory, inhibitory control, sustained attention, mental health.	Lifelong. Mean of six-month median Phe levels.	Higher Phe levels correlated with poorer working memory, inhibitory control and sustained attention.	Not specifically addressed in the statistical design. The relationship between childhood, adulthood and lifetime Phe levels and cognitive outcome measures was assessed for each of these three periods separately.	Normal IQ distribution (100 +/- 12).	None
Romani 2017 [15]	37	Early-treated adult patients.	Mean 27.5 SD 7.3	Visuospatial attention, visuomotor coordination, complex executive functions, inhibitory control, short-term/working memory, sustained attention, orthographic language, spoken language, verbal memory and learning.	Lifelong averaging median annual Phe levels, split into childhood (0–10), adolescence (11–16) and adulthood (17+).	Higher Phe was associated with lower IQ and worse performance on all domains except inhibitory control.	Visuomotor coordination, sustained attention, and verbal memory and learning remain significant when adjusting for childhood Phe.	Mean percent of abnormal scores was 13.2% (15.1 SD).	None

A second study in a smaller cohort of early treated adults with PKU (*n* = 57, comparison to matched healthy control subjects, treatment started before 3 months of age) found a negative relationship between Phe levels on the day of testing and attention tasks, which remained significant when historical Phe levels were taken into account [14]. third study by Romani et al. examined cognitive performance in 37 early treated adults with PKU compared to 30 control subjects matched for age, gender and educational status, with information on average Phe levels in childhood, adolescence, adulthood and Phe at the time of testing [15]. When average Phe level in adolescence was included in the model, the positive relationship between Phe levels at time of testing with worse scores on memory, sustained attention and visuo-motor tasks remained significant [15]. The authors concluded that visuospatial attention is best predicted by childhood metabolic control (both Phe average and variation), and executive functions by adolescent Phe average as well as by childhood Phe fluctuation. However, visuomotor coordination, sustained attention, and verbal memory and learning are best predicted by current Phe levels, but also impacted by Phe fluctuations across a lifetime.

An additional way of assessing the outcome of early treated PKU in adulthood, in order to answer question 1, is to investigate the proportion of early treated adults with normal neurocognitive scores. In the Aitkenhead et al. study, 53% (79/147) of the cohort, including 60 individuals whose Phe was > 600 µmol/L on the day of testing (cohort range 119–2135 µmol/L) achieved normal scores (above − 1.5SDs of the normative sample mean) in all seven cognitive function tests [5]. It is important to note that in the general population only 69% of all tested individuals would be expected to have no impaired scores (i.e. normal scores on all 7 tests). In a study by De Felice et al., in which language processing and executive functions were assessed in 15 to 33 adult etPKU patients (numbers differed between the tasks performed), 50% had completely normal scores (average Phe on the day of testing 751 ± 319 µmol/L) [16] The studies by Romani et al. (2017) and Palermo et al. (2017) reported on the same 37 early-treated adults with PKU (Phe on the day of testing 720 ± 343 µmol/L, range 66-1465), of whom 23 (62%) had no abnormal results in any of 36 cognitive measures assessed [13]). Christ et al. (2020) reported data from 128 early treated adults (Phe closest to time of testing: 771 ± 457 µmol/L, range 78-2024) who completed the adult (self-report) versions of the Behaviour Rating Inventory of Executive Function (BRIEF) tool. For each individual component of the BRIEF, at least 70% of participants scored in the normal range [17].

In summary, a proportion of adults with etPKU perform worse in several domains on cognitive function testing compared to matched control subjects. The older the studies, the worse the performance, likely indicating that PKU treatment has improved over time from the late 1960s onwards. Neonatal, childhood and adolescent metabolic control have a clear influence on cognitive functioning in adulthood. Phe levels measured at the time of testing in adulthood are independently related to processing speed. However, for the other domains an effect independent of current Phe levels cannot be established with the currently available data as discussed by Thomas et al. where the authors attempted to address this question, but could not run meta-analyses for the different domains because of a lack of reported quantitative data [18]. Overall, these studies show that residual PKU is an ill-defined combination of abnormalities on neurocognitive testing, not uniform across the full cohort of etPKU patients. Its relationship to historical childhood Phe is clear. In contrast, Phe in adulthood is only clearly associated with processing speed.

The studies described above are all cross sectional, describing cognitive functioning in etPKU adult patients at a single point in time. To date, there is only longer-term longitudinal study reporting cognitive outcomes in adults with PKU. 57 patients (age range at baseline: 19–41 years; mean age 31 years) were followed up at 5 years [19]. 35 of the original cohort returned at 10 years and 27 at 15 years for verbal and performance IQ testing (WAIS III), a Trail Making Test (information processing speed), and a d2-test (sustained attention) [20, 21]. There was no change in any of the test outcomes at either 5, 10 or 15 years follow up. This stability is observed despite the fact that the Phe levels in adulthood were above the recent American recommended upper level (< 360 µmol/l) in all patients and above the European (< 600 µmol/l) in a significant proportion of patients (Phe levels on the day of testing: 912 ± 516 µmol/l in patients < 47 years old and 1002 ± 540 µmol/l in older patients). There was stable mild impairment in older PKU patients, related to high Phe levels in adolescence as a consequence of early relaxation of the protein restricted diet.

### Well-being in early treated adults

Studies discussed in this section are summarized in Table 2. A variety of ‘well-being’ measures including anxiety and depression scales, social functioning, health-related and PKU-related quality of life have been reported in early treated adults with PKU. These measures have been assessed regarding their relationship to Phe (either as current Phe and/or as an average Phe measurement in the years preceding testing).

**Table 2:** The association between Phe levels and mental health and quality of life in adults with PKU

First author Year	Number of patients	Type of patients	Age at assessment	Assessments	Periods for which Phe levels are analysed	Differences between PKU patients and healthy control subjects	Relationship between Phe levels and mental health/social functioning	Funding by pharmaceutical or nutritional company of study or employment of author(s)
Bosch 2007 [22]	32 patients; 508 controls	etPKU	24.6 *±* 3.6 years	Course of life questionnaire; RAND 36 Quality of Life scale	Not analysed	No difference between PKU and controls on course or quality of life measures	Not analysed	SHS International/none
Bilder 2013 [23]	64 PKU	Not defined	26 *±* 7.2 years	Brief symptom inventory	Average Phe level in 2 years prior to study and recent Phe level (within 1 month of study)	No control groups so measured against norms developed for the screening tool; 50% of participants had a positive screen on BSI	Average Phe in 2 years prior correlated with positive symptom distress inventory and psychoticism but no other parameters	BioMarin Pharmaceutical Inc/none
Clacy 2014 [24]	8 PKU	“Classical” PKU; no other details	19.4 *±* 3.6 years.	Depression and anxiety stress scale	Concurrent level (day of testing) Lifetime levels which are not controlled for changes in monitoring frequency with age (so weighted towards control in childhood)	No active controls; results compared to population norms but no statistical analysis provided to determine in PKU patients differed from population norms	Lifetime Phe significant correlation with anxiety and stress but not depression; lifetime ratio of Phe to tyrosine correlated with depression, anxiety, stress; No correlation of concurrent PHE with outcomes; concurrent PHE to tyrosine ratio correlated with stress	None/none
Bosch^1^ 2015 [25]	306 patients (104 adults); 253 parents	etPKU and parents	Adult sample 25.8 *±* 6.6 years	Pediatric Quality of Life Inventory, Child Health Questionnaire; PKU QoL, SF36	Not analysed	Highest impact scores were anxiety about Phe levels during pregnancy and emotional impact of poor adherence to diet; better physical scores than normative populations	Not analysed	Merck Serono involved in study conduct and data review/ 1 author employed by subsidiary of Merck
Jahja^2^ 2016 [26]	95 PKU and 95 controls	etPKU	21.6 *±* 10.2 years	Social skills checklist self-report; Amsterdam Neuropsychologic Tasks - Facial Recognition, Identification of Facial Emotions; Faux-Pas Recognition Test, Reading the Mind in the Eyes task	Concurrent (day of testing) Lifetime – mean of half-year median Phe levels Mean at other developmental intervals – 0–7, 8–12, 13–17, *≥* 18 years	No differences between PKU and controls for social skills in children < 12 years; social skills worse in PKU than controls in those *≥* 12 years although effects reduced (but still significant) when controlled for age and IQ	No associations between concurrent Phe and outcome measures in any age group. Lifetime Phe associated with social performance for those *≥* 12 years; in adults, social skills correlated with Phe *≤* 12 years but nonsignificant correlation with levels 13–17 years	None/none
Jahja ^2^ 2017 [14]	57 PKU patients and 57 controls	etPKU	27.7 *±* 6 years	Adult Self-Report (ASR) of the Achenbach System of Empirically Based Assessment	Concurrent level (day of testing) Lifetime level – mean of half-year median levels Childhood level – mean of levels between 0–12 years	Higher levels of self-reported depressive and avoidant personality problems than age-matched controls	No correlation between any measure of PHE and mental health outcomes in full group; more symptoms on depressive and avoidant personality scores than controls with those with lifetime Phe > 360; depressive symptoms also more common than controls in those with childhood Phe > 360	None/none
Huijbrechts^2^ 2018 [27]	90 PKU and 109 controls	etPKU	21.0 *±* 10.1	TNO-AZL questionnaire for health-related quality of life – parents completed scale for children < 12; age-appropriate questionnaires completed by patient for other age groups	Concurrent PHE (day of testing); lifetime PHE (obtained through review of clinical records)	No differences in HRQoL for patients relative to controls for those 15 years and under; adults with PKU more likely to report depressive mood, anger, and pain.	Lifetime Phe negatively correlated with sleep, pain, sexuality, and anger in adults only, no correlations in younger patients. Concurrent Phe negatively correlated with sexuality in adults.	None/none
Barta 2020 [28]	88 PKU	etPKU	31 (range 25–40) years;	PKU-QoL questionnaire	Mean PHE from previous year; mean PHE from past 10 years, “lifetime PHE” for some patients; concurrent PHE	No controls so compared with norms related to the assessment tool; No domain with major impact on symptoms; Mild to moderate impact seen for 6 domains, 4 of which were related to the diet itself – e.g. Guilt if nonadherent to diet or supplements, taste of supplements.	Concurrent and 1 year Phe related to anxiety about Phe levels; 10-year Phe related to emotional impact of PKU	None/none
Palermo 2020 [29]	36 PKU; 40 controls	etPKU	27.4 *±* 8.3	Beck Depression Inventory-II Interpersonal Reactivity Index Mental Health Quality of Life scale from SF-36, Comprehensive Affect Test System; Likert scale to rate perceived ease of remaining on diet	Average PHE level in different age bands: 0–10 years, 11–16 years, 17 years to present; concurrent PHE level	No differences from controls on any mental health or emotional health tests; Perceived ease of staying on diet negatively correlated with BDI and positively with emotional well-being on SF-36.	Average Phe in bands 0–10 and 11–16 years but not 17 years and older correlated with some scores on empathy scale. Concurrent Phe only correlated with personal distress.	None/none
Aitkenhead 2021 [5]	154 PKU 76 controls	etPKU	32.4 *±* 8.9	Mental health component of SF-36, Hospital Anxiety and Depression Scale; Perceived Stress Scale, Relationship Scales Questionnaire; Family Adaptability and Cohesion Scale	6-month median PHE for age bands: 0–6, 6–12, 12–18 and > 18 years; Defined groups based on control < 12 and > 18: low/low; low/high; high/high	No differences from controls on emotional, or social functioning but PKU patients more likely to have “anxious relationship style” than controls.	Partially adherent to diet group had lower mental and physical quality of life than those adherent or non-adherent. No differences in psychosocial outcomes in on/off diet groups and no differences in psychosocial outcomes for those well controlled in all age groups vs. those well controlled as children with poor control as adults. High/high group tended to have lowest scores for anxiety although not large enough for statistical testing	Nutricia/none
Altman 2021 [30]	244 PKU	Mix of etPKU and patient born before newborn screening	35.9 years	Single centre retrospective chart audit looking for key words: depression, anxiety, low mood, mood swings	Average PHE in previous 2 years	No normal controls so unable to compare; prevalence of mental health symptoms was 33.3% in those born before NBS and 16.3% in those born after NBS.	Compared to those with average Phe < 600 umol/L, those > 600 umol/L more likely to have chart record of depression and low mood and trends to higher rates of reported anxiety and mood swings.	None/none
Maissen-Abgottspon 2023 [31]	124 PKU	Early treated classical PKU	30 (range 22.5–37.6)	PKU-QoL; Beck Depression Inventory II	Concurrent PHE, mean PHE of past one year	No controls so compared with norms related to the assessment tool; Fatigue one symptom with moderate impact; females more impacted than males due to anxiety about PHE during pregnancy; single site subgroup analysis significant relationship between perceived overall impact of PKU on self-reported QoL and depression score	Higher concurrent and 1 year Phe levels associated with higher perceived impacts of protein restriction on QoL.	None/none.
Boland 2024 [32]	40 PKU 40 controls	etPKU	26.0 *±* 6.0 years	Adult Self-Report and Adult Behaviour Checklist from Achenbach System of Empirically Based Assessments; Mini International Neuropsychiatric Interview	Concurrent PHE level; mean PHE for year prior to assessment; lifetime PHE level – half year median level for 0–5, 6–11, 12–17 and > 18 years.	Nonsignificant trend to more anxiety in PKU than controls	No relationship of concurrent PHE or lifetime PHE to anxiety;	None/none

A high number of adults with PKU are lost to follow up [33–35] and this confounds interpretation of the data on mental and emotional health outcomes more than any other PKU related outcome. Individuals who perceive challenges in these areas may be more likely to seek health care. Even with this caveat, the results relating PKU and/or Phe levels to psychiatric symptoms are inconsistent [36]. Several studies show no correlation between Phe in adulthood and measures of depression, anxiety, mental health or social functioning [14, 24, 26, 29]. The largest study to date in early treated adults showed comparable educational, occupational, emotional, and social functioning outcomes in PKU and healthy controls [5]. Multiple studies suggest that most early and continuously treated adults had a quality of life (QoL) comparable to their peers or to the general population [22, 25], with more than 75% of participants rating their health related QoL as good or better [28, 31].

Other studies have, however, found associations between Phe and measures of mood, anxiety, or health-related QoL in adulthood [23, 27, 32, 37, 38]. A UK single centre chart review suggested that patients with Phe levels above 600 umol/L were more likely to have a chart record of low mood than those with lower levels [30]. A Hungarian study found modest correlations between both concurrent and long term Phe levels and PKU-QoL scores [28], whereas a Dutch study found only lifetime Phe, not concurrent Phe, to predict symptoms of depression and anger on multivariable analysis [26]. Recent (2 year) Phe levels were related to psychiatric symptoms relative to population norms in the USA based ADAPT study, but the high rate (50%) of positive screens on the Brief Symptom Inventory at study entry may reflect an ascertainment bias [23].

While data are mixed on relationship between Phe levels in adulthood and QoL outcomes, data on the impact of the PKU diet on QoL are more consistent. Adults who are partially adherent to diet have been shown to have worse emotional health measures than adults who are either fully adherent or fully off diet [5, 28]. It is difficult to determine cause and effect for this observation: Good adherence may lead to positive changes in quality of life and/or less guilt or, alternatively, these results could simply reflect that good subjective health status facilitates therapy adherence [5]. Adults with better childhood metabolic control may exhibit more anxiety compared to those with less tight control [5, 29]. The reasons for this are unclear, but one hypothesis is that parental anxiety leads to lower Phe concentrations in childhood, but also to a more anxious relationship style in adults with PKU later in life [5]. The same studies also showed that anxiety is higher in groups with lower concurrent Phe levels, with an inverse correlation between Phe levels and measures of emotional and mental health [5, 29]. Another large multicentre study showed the impact of protein restriction on QoL was highest in those with the highest Phe levels, suggesting that some adult patients struggle with guilt when diet adherence is incomplete [31].

### Neurological complications in adults with early-treated PKU

There are multiple reports of adults with PKU having brisk reflexes and / or a mild-moderate upper limb tremor on examination [39–42]. For the majority of adults these findings have limited functional consequences. A small number of case reports and case series have reported late-onset neurological complications in adulthood thought to be related to PKU [43–47] (results summarized in Table 3) – predominately ataxia, pyramidal signs with spasticity, seizures or visual loss. These complications have recently been summarized [48]. Several of these patients had suboptimal PKU treatment in childhood / adolescence, and/or poor nutritional status in adulthood – with vitamin deficiencies including B12, folate and vitamin D. Three patients had discontinued PKU diet before the age of 10 years. Phe (when reported) was > 1000 µmol/L at the time of reported neurological complications in all individuals.

**Table 3:** Neurological^#^ complications in individuals with early treated* PKU

Author, Year	M/F, Age (years) at complication onset	Age at stopping PKU diet/Phe at time of neurological presentation	Complication	Symptom resolution (over time period)	Phe at time of / after resolution	Other issues reported
Thompson, 1990 [70]	F, 13	Not reported/ 500–1300 umol/L	Seizures; ataxia; brisk lower limb reflexes	Not reported	Not reported	Suboptimal childhood Phe: 400–700 umol/L to 10 years; 500–1300 umol/L thereafter.
Thompson, 1990 [70]	M, 22	19 years/ 900–1600 umol/L	Ataxia; brisk reflexes	Not reported	Not reported	Suboptimal childhood Phe: 600–1200 umol/L, 8–10 years; 900–1600 umol/L thereafter.
Thompson, 1990 [70]	M, 16	18 years/not reported	Seizures; behavioural issues; brisk reflexes	Not reported	Not reported	Diet started at 6 weeks. Suboptimal childhood Phe: 600–800 umol/L 10–14 years; 900–1100 umol/L thereafter.
Thompson, 1990 [70]	M, 18	16 years/ 1334 umol/L	Hyperreflexia and pyramidal weakness of legs	Not reported	Not reported	Diet started at 7 weeks.
McCombe, 1992 [47]	M, 19	18 years/ 1900 umol/L	Spasticity and pyramidal weakness of 4 limbs	Near complete (6 months)	1760–2410 umol/L	Vitamin B12 deficiency also treated.
Rubin, 2013 [49]	M, 25	23 years/ 1512 umol/L	Bilateral visual loss; central scotoma	Complete (1 month)	120–300 umol/L	
Anwar, 2013 [45]	F, 41	16 years/ 1564 umol/L	38 years – bilateral visual loss; 41 years – spasticity and pyramidal weakness	Partial (2 months)	121 umol/L	Vitamin B12 and folate deficiencies.
Jaulent, 2020 [46]	M, 19	19 years/ 1710 umol/L	Cerebellar syndrome	Complete (few days)	600–1100 umol/L	Premature birth (26/40).
Jaulent, 2020 [46]	F, 37	7 years/ 1310 umol/L	Cerebellar syndrome	Partial	800–1600 umol/L	Suboptimal childhood Phe: 600–1200 umol/L.
Jaulent, 2020 [46]	F, 41	8 years/ 1160 umol/L	Transient right hemibody parasthesia; aphasia; headache	Partial	800–1000 umol/L	
Jaulent, 2020 [46]	M, 29	29 years/ 1330 umol/L	Unilateral visual loss; paracentral scotoma	Complete (12 months)	800–1000 umol/L	
Jaulent, 2020 [46]	F, 33	18 years/ 1980 umol/L	Bilateral visual loss; cerebellar ataxia; paraparesis	Complete (6 months)	300–1000 umol/L	
Jaulent, 2020 [46]	M, 33	8 years/ 1320 umol/L	Bilateral visual loss; optic neuropathy; cerebellar syndrome; dysexecutive syndrome	Partial (6 months)	900–1000 umol/L	Vitamin B9, B12 and C deficiencies.
Merkel, 2023 [48]	F, 50	14 years/ 1816 umol/L	Ataxia and spastic paraparesis	Complete (6 months)	726–969 umol/L	Vitamin B12 and D deficiencies.
Merkel 2023 [48]	M, 41	17 years/ 1271 umol/L	Bilateral visual loss; tetraspasticity	Near complete (6 months)	1029 umol/L	

^#^Includes only those individuals with visual, gait and balance issues. Articles reporting isolated signs including tremor and brisk reflexes are not included. *Diagnosed by screening and started on a Phe-lowering diet within the first 8 weeks of life

Five patients who presented with acute visual loss all had Phe > 1300 µmol/L [45, 46, 49]. Phenylalanine lowering treatment was intensified and three of the five had complete resolution of their symptoms (two had partial resolution), though in some cases Phe remained elevated at up to 1000 µmol/L.

Due to the rarity of reports of acute neurological compromise in early treated adults with PKU it remains difficult to be sure if these are a definite complication of hyperphenylalaninemia or related to an additional underlying diagnosis. Another potential contributing factor could be nutritional deficiencies such as vitamin B12 deficiency. All the cases reported to date have improved clinically (some with complete resolution) with reintroduction of a restricted protein diet and nutritional supplementation, suggesting the potential for reversibility of these complications.

### Interventional studies to alter Phe in adults with PKU – effects on cognition, mood and other outcomes

Several studies have attempted to either decrease or increase brain Phe availability and measure the effect on outcomes in adulthood. Phe manipulation has been attempted by dietary interventions [50–53]), BH4 treatment [54, 55], large neutral amino acid supplementation [56–58], oral phenylalanine loading [59–61] and Pegvaliase treatment [62–64].

#### Dietary interventions aimed at reducing Phe levels

The four dietary intervention studies are small (7, 9, 15 and 16 participants respectively) and all are (unavoidably) unblinded [50–53]. Results are summarized in Table 4. Apart from the small sample size and the lack of appropriate controls, other limitations of the studies are: the high number of variables tested (in the low number of participants) [51, 53], patient sample not representative of etPKU patients in general (e.g. not treated throughout adolescence) [51] and inconsistent results (better performance with lower Phe levels, but this was maintained after Phe increased again) [50]. Overall, no firm conclusions of the effects of dietary interventions lowering Phe levels in adult etPKU patients on cognitive functioning and mood can be drawn from these studies.

**Table 4:** The effects of dietary Phe lowering/increasing Phe interventions in individuals with early treated PKU

First author Year	Number of patients	Type of patients	Age	Intervention	Duration	Outcome Measures		Baseline Phe (umol/L)	Phe after intervention	Outcome	Study funding by pharmaceutical companies/ Authors employed by pharmaceutical companies
**Phe lowering**											
Burgess 2021 [51]	9	Early treated	Mean 33 (SD 8.8)	Re-initiation of diet	12 months	Objective cognitive function, anxiety and depression scale scores		Mean 1108 (SD 293)	Only mean delta Phe given: -372 (SD 250)	Changes in cognitive performance index, Hamilton Anxiety and Depression Rating Scales, Processing speed index and Working memory index, but no significance given for the changes. No clear relationship between changes in Phe and these changes.	Biomarin Pharmaceuticals provided funding for open access publication fees/None
Schmidt 1996 [50]	15	Early treated, normal IQ	Mean 21 (range 17–24)	Strict Phe reduced diet	4–5 weeks each phase (regular diet-low Phe diet-regular diet)	Attention		Mean 1320 (range 720–1800)	630 (range 280–966)	Significant improvement in the attention task with lower Phe levels but this effect remained after Phe increased again, non-significant increase in calculation speed with lower Phe	None/None
Dawson 2011 [52]	16	NBS diagnosed, early treated	32.2 (SD 5.2)	Pre-conception diet	NS	Saccadic latency		1223 (SD 330)	277 (108)	Significant lower median latency with lower compared to higher Phe	None/None
Manti* 2023 [53]	7	NBS diagnosed, early treated, suboptimal control during childhood and adolescence	31.1 (SD:1.6)	Pre-conception diet	6 months (assessments at baseline, 3 and 6 months)	Neurocognitive testing assesing IQ, sustained and visuospatial attention, inhibitory and visuomotor control, (working) memory and learning		1311 (SD 278)	166 ± 64 at 3 months and 162 ± 51 at 6 months	Significant improvements reported in all domains except language	None/None
**Increasing Phe levels**											
Ten Hoedt 2010 [59]	9	NBS diagnosed, early treated	Range 19–34	Orally administered Phe** or placebo, randomised double-blind placebo-controlled	4 weeks each phase (Phe and placebo)		Neurocognitive testing^$^ and Profile of Mood States (POMS) questionnaires	790 (SD 332)	Phe loading: 1259 (SD 332) Placebo: 709 (SD 322)	Significant worse scores for several aspects of mood in the in the Phe loading versus placebo phase (more fatigued and less vigorous). Significant more fluctuation in tempo during the Phe loading versus placebo phase. No significant changes in all other neurocognitive testing domains	Nutricia Advanced Medical Nutrition provided the funding for this study/None
Sundermann 2011 [60]	17	NBS diagnosed, early treated	31.0 (SD 5.2)	Orally administered 100 mg L-Phe per kg body weight or a placebo (single dose)	Two single doses (placebo or Phe) 21.6± 2.1 days apart		An adapted color-word matching Stroop task	1180 (SD 320)	Phe loading: 2170 (SD 320)	No impact of the increase in Phe levels as a result of Phe loading on reaction times and error rates	None/None
Trepp 2023 [61]	30	NBS diagnosed, early treated	Median 36 (range 19–48)	Orally administered Phe** or placebo, double blind in randomized order	4 weeks each phase (Phe and placebo)		Neurocognitive testing and questionaires (POMS/Beck depression inventory)	Group 1: median 733 (range 466–1084) Group 2 median: 750 (range 380–1208)	Phe loading group 1: median 1455 (IQR: 1228–1746) and group 2: 1457 (IQR: 1373–1698)	No impact of the increase in Phe levels as a result of Phe loading on the neurocognitive test results nor on the mood questionnaire results	Nutricia Metabolics Research Fund/None

*the results of this study are not line with those of other studies. In particular, the baseline IQ is lower than average compared to other etPKU populations (most likely explained by poor childhood and adolescent metabolic control) and the improvements are over all domains tested, even IQ. This suggests a learning effect (despite efforts to control for this) **amount of Phe representing the amount of Phe ingested whilst following a normal diet for a healthy individual, thus sex and weight dependent ^$^ simple reaction time, sustained attention, visuospatial processing, working memory capacity, inhibition and set shifting and visuomotor control (random pursuit, circle tracing) ^#^working memory, accuracy, reaction time, sustained attention, manual dexterity, anxiety, vigor, fatigue, anger, depression

#### BH4 treatment

Burton et al. (2015) showed no significant improvement in ADHD Self-Report Scale total, the Inattention and Hyperactivity/Impulsivity Subscale scores, in 12 adults with ADHD symptoms randomised to BH4 or placebo for 13 weeks [55]. In the same study, 53 adults who responded biochemically to BH4, completed a self-reported BRIEF questionnaire, with again no differences in the BH4 and placebo-treated groups at 13 weeks follow up. Five adults (none of whom were biochemically responsive to BH4) were treated for 4 months with BH4 at 20 mg/kg/day [54]. A battery of 12 neuropsychological tests was performed at baseline and after 4 months treatment. No significant differences in test results were recorded.

#### Large neutral amino acid supplementation

Scala et al. (2020) reported on 10 adults who were treated with LNAA supplementation (0.8–1.0 g/kg/day divided in three doses) for 12 months, with neuropsychological testing at baseline, 3 and 12 months [56]. There was no change in plasma Phe during LNAA supplementation, but plasma tyrosine increased significantly. There were no differences in hand dexterity (9-hole PEG test) at 9 months, but improvements were noticed in general well-being (non-significant), some executive functions (Wisconsin Card Sorting Test) and attentional performance (TAP test).

#### Phe loading

In studies using Phe loading a standardised amount of phenylalanine is given, in the form of capsules, to patients with a known baseline Phe to study the effect of a substantial increase in Phe levels. Studies discussed in this section are summarized in Table 4. In the largest double blind study performed to date, 30 adult early-treated PKU patients on dietary treatment (median baseline Phe 749, range 380–1208) were given either placebo or 1500–3000 mg Phe per day over 4 weeks, in a randomised order [61]. The primary endpoint was working memory accuracy and additional cognitive domains, mood, and depression served as secondary endpoints in a non-inferiority analysis for Phe versus placebo. Phe intake was found non-inferior to placebo with respect to working memory accuracy, working memory reaction time, manual dexterity, mood, and depression. More adverse events (AEs) were observed during the Phe than during the placebo period, though only in the first randomisation phase and most AEs were deemed not related to Phe administration.

Two smaller studies were performed (*n* = 9 and *n* = 17) to investigate the impact of Phe loading. The first, a 4 week double blind randomised placebo controlledtrial by ten Hoedt et al. showed a negative effect of Phe loading on the fatigue and vigor subscales of the Profile of Mood States) questionnaire. Only one measure (fluctuation in tempo of the sustained attention task) of the Amsterdam Neuropsychological Tasks worsened significantly following Phe loading compared to placebo (F_1,8_ = 7.02, *p* = 0.029, power = 0.64) [59]. In a second study a single Phe load (100 mg/kg) did not result in a difference in reaction time or error rates in a color-word matching Stroop task compared to placebo [60]. In summary, increasing Phe levels up to 4 weeks does not lead to significant changes in cognitive functioning, nor in depressive feelings in etPKU patients.

#### Pegvaliase treatment

Pegvaliase (Palenziq^®^) is an enzyme substitution therapy in which a pegylated derivative of the enzyme phenylalanine ammonia-lyase is administered subcutaneously to metabolize phenylalanine. There are no large cohort studies with a comprehensive neuropsychological test battery performed before and during treatment with Pegvaliase and the available publications lack justification of the specific subset of cognitive/psychological tests they chose to report. Details of the methodology and outcomes of the two relevant studies (Harding et al. and Thomas et al.) [18, 62, 63] are given in supplementary file 1. Harding et al. reported on 86 adults previously treated with Pegvaliase, of whom one third discontinued Pegvaliase treatment for 8 weeks, which did not lead to deterioration in neuropsychiatric or neurocognitive symptoms (despite a significant increase in blood Phe concentration from < 600 µmol/L to > 1000 µmol/L). Part of the Cambridge Neuropsychological Test Automated Battery (CANTAB) was administered in 9 patients only, which did not result in a meaningful comparison (6 Pegvaliase versus 3 placebo treated patients).

The report by Thomas et al. [62], with further details of outcomes of the same paper by Bilder et al. [64], shows reduction on the inattention subscale of the ADHD RS-IV score in 261 adults that started treatment with Pegvaliase, 169 of whom completed a long-term extension study. Interestingly, this improvement was also observed in the quartile of patients in which no reduction, or even an increase, in plasma Phe was observed, suggesting this was an effect of clinical trial participation itself rather than the actual effect of PKU treatment. The smallest intervention study reporting on cognitive functioning before and during treatment with Pegvaliase by Burlina et al. was conducted in only 3 patients and is therefore not discussed here [65].

In conclusion, intervention studies to alter Phe in adulthood have shown variable results. The largest placebo-controlled intervention study increasing Phe levels did not show worsening of cognitive functioning, for the domains tested, with higher Phe levels. Whether attentional symptoms and performance may improve with lowering of Phe concentrations needs to be confirmed in larger placebo-controlled trials of longer duration and using validated neuropsychological test batteries (not automated ones) with corrections for multiple testing.

## Discussion, recommendations and conclusions

### Cognition, neurological functioning, quality of life and their relationship to Phe levels

Here we come back to the questions posed in the introduction, which we will briefly answer in this section.

Question 1: What is the cognitive profile of early treated adults with PKU and how does this relate to concurrent and historical Phe levels. Residual PKU can be defined as below average functioning on several neurocognitive domains. This affects a proportion of etPKU patients, with significant heterogeneity in the level of impairment as well as the specific domains involved. Residual PKU is clearly related to higher than recommended Phe levels in childhood and adolescence. Only processing speed has been shown to be independently related to Phe levels at the time of testing, though again with great variability between individuals. For other domains, no clear relationship with Phe in adulthood and/or the day of testing has been shown after correcting for historical Phe levels.

Question 2: What is the current quality of life of early treated adults with PKU (including mood, educational, occupational, and social functioning) and how does this relate to concurrent and historical Phe levels? Most studies show normal (or near normal) health related and general QoL scores in early treated adults with PKU. Phe control in childhood has a complex relationship with adult QoL outcomes. Evidence for a relationship between Phe concentrations in adulthood and abnormal QoL findings is mixed. Data do suggest that incomplete adherence to PKU dietary recommendations negatively impacts emotional well-being and QoL when compared to full adherence or no dietary treatment at all. This risk needs to be considered when making any recommendation as to treatment targets in adults. Taking into account the negative effects of dietary treatment in a subgroup of etPKU patients, individual treatment targets may change once non-dietary treatments with a favourable side effect profile become available.

Question 3: Is there evidence for new onset progressive neurological or cognitive disease in early treated adults who discontinue or relax phenylalanine lowering treatment in adulthood? To date there are no long-term follow up data to suggest that high Phe levels in adults with etPKU lead to progression of neurocognitive impairments. There are limited case reports of new onset neurological symptoms that might be related to high Phe and/or dietary deficiencies that generally improve upon reinstitution of dietary treatment.

Question 4: Do interventions to alter Phe concentrations in adulthood change cognitive, occupational and social functioning? There are no data which convincingly show improvement of cognitive functioning with lowering of Phe levels in adulthood.

### Recommendations on treatment targets

Question 5: Should there be a recommended Phe concentration for all non-pregnant adults? Or should it be different for different (age) subgroups? Based on the findings discussed above, at this moment in time, there is not sufficient evidence to support any recommended target Phe level for all adults with etPKU. Maintaining plasma Phe < 600 umol/L up to (at least) age 20 years seems sensible given the results of the Feldman et al. long-term follow up studies showing significantly worse cognitive function in older adults who have had higher Phe levels between age 11 and 20 years [19, 21]. In later adulthood, a patient-centred approach to optimize nutritional status and support the setting of individualised treatment goals should be taken. Any patient who wishes to maintain phenylalanine levels (e.g. < 600 umol/L) because they wish to avoid *potential* long term negative consequences of higher levels, should receive full support.

If patients report deficits in mood or cognition these should be objectively defined and quantified as much as possible (using questionnaires and neuropsychological / neurocognitive examinations) and the relationship between the occurrence of these symptoms and high Phe levels should be established before treatment is intensified. Many of the symptoms attributed to high Phe levels (e.g. brain fog, impaired concentration etc.) are very common in the general population and the number of adults reporting subjective cognitive impairment has risen over the years [66]. Next, improvement with lower Phe levels should be documented and if improvement occurs a personal target Phe level should be established (e.g. <600, < 800 or < 1000 umol/L). Support to reach these individuals treatment goals includes (but is not limited to) access to reliable information, support in executing dietary treatment, reimbursement of medical foods and access to a treatment team including a physician trained to take care of adult patients, as well as a metabolic dietician. In BH4 responsive patients, BH4 can be added to the treatment, provided there is a significant benefit and a sufficient diet is ensured, with emphasis on adequate total protein and vitamin/trace element intake.

### The role of pegvaliase in adult etPKU treatment

Pegvaliase treatment can be considered, but the considerable side effects (such as arthralgia and injection-site reactions), the high rate of discontinuation, the risk of iatrogenic hypophenylalaninemia and the risk of suboptimal total protein intake [67–69], which can potentially result in nutritional deficiencies, should be weighed against the uncertain long term health benefits of reaching lower Phe levels. In addition Pegvaliase treatment does not always ensure freedom from dietary treatment [69].

### Discontinuation of Phe lowering treatment

Patients who do not wish to achieve a specific phenylalanine target level, after having had the opportunity to discuss the currently available evidence on outcomes, should not be pressed to do so. They should continue to be followed in the adult metabolic outpatient clinic to monitor general wellbeing and potential PKU/diet related complications (such as iron/B12 deficiency, reduced bone mineral density, obesity etc.). They should be provided with education about the importance of follow-up, with an emphasis on achieving a nutritionally complete diet, as well as monitoring for difficulties in social and executive functioning and the occurrence of neurological signs and symptoms. If at any point in time they wish to change their treatment goal and return to dietary treatment, they should receive support to do so. In any adult who is not on treatment, or in whom metabolic control is considered suboptimal, acute neurological or visual deterioration should prompt standard investigations for an underlying cause and discussion. The chance of developing these complications is higher in etPKU patients who had suboptimal Phe levels in childhood and/or discontinued dietary treatment in adolescence (Table 3). If no other cause can be identified, phenylalanine lowering treatment with specific nutritional supplementation should be reintroduced. The acute neurological symptoms are (partially) reversible in the majority of patients and it is currently unclear whether this reversibility is due to lower Phe levels, correction of nutritional deficiencies or a combination of both.

### Future perspectives

The coming years, in which new PKU treatments will be assessed in clinical trials, will provide great opportunities to study the effects of lowering Phe levels in adults with early-treated PKU over longer periods. We would suggest that regulatory authorities mandate a robust set of ‘before and after’ neurocognitive and psychosocial measures when new treatments are being assessed in adults. However, as pharmaceutical trials are limited to a relatively short period of time in the lifespan of patients, there is a need for long-term publicly funded data collection from birth to at least middle age. A large, independent, multicentre registry is needed in order to study the potential correlation between Phe levels at the different stages of life and the neurological and cognitive functioning of etPKU patients. The high costs of some therapies mean that public funding of such a register would be cost effective. Finally, there is a clear need for long term monitoring of cognitive functioning in all adults with PKU using a standardised neuropsychological test battery at intervals (e.g. every 5 years) throughout adult life and resources to achieve this goal must be provided to the adult clinics following such patients. We refer to Sect.  7.5 of the revised European PKU guideline for a recommended test battery relevant to PKU [2].

### Limitations of the data and the analysis

We have discussed the currently available evidence about residual PKU and its relationship to current and historical Phe levels. There are several factors complicating evidence analysis: (1) There is a lack of long-term follow-up and intervention studies in large cohorts of individuals with etPKU. (2) Patients with low Phe levels in adulthood are more likely to have had optimal control earlier in life, which confounds interpretation of the benefits of lower Phe levels in adults. (3) Adults who are ‘off-diet’, with higher Phe concentrations, may also have diets of poorer quality with low protein intake and secondary dietary deficiencies (e.g. vitamin B12) that may contribute to their phenotype. (4) Given that NBS only started to become more widespread since the 1970s, the reported number of early-treated individuals over the age of 50 years is still quite low. Finally, (5) the high rate of loss to follow up in adult patients with PKU means that those data that have been published may not reflect the experience of the patient population as a whole [33].

## Summary

If treated well in childhood, educational, occupational and social outcomes, are good in adults with PKU. Continuous treatment aimed at lowering Phe levels should be made available to all patients, treatment benefits should be objectified and well documented. Some patients will report that they feel better with lower Phe concentrations. On the other hand, if an individual patient feels well, has a nutritionally complete diet, understands the uncertainties regarding long term outcome, but does not feel any specific benefit of Phe lowering treatment for them as an individual – then they should be offered monitoring as a supported alternative. Lifelong monitoring and nutritional support is key for all patients.

## Electronic Supplementary Material

Below is the link to the electronic supplementary material.

Supplementary Material 1

## Data Availability

The dataset(s) supporting the conclusions of this article is(are) included within the article (and its additional file(s)).

## Abbreviations

Phe: Phenylalanine
etPKU: Early treated phenylketonuria
RT: Reaction time
AE: Adverse event
