# Supplementary material for: Treatment goals for adults with early treated PKU should be determined by evidence-based shared decision making between patients and their medical team
Source: Orphanet J Rare Dis. 2026 Jun 2;21:233. doi: 10.1186/s13023-026-04376-5 (PMC13307702; doi:10.1186/s13023-026-04376-5)
Supplement: Supplementary file 1 — Supplementary Material 1 [file 13023_2026_4376_MOESM1_ESM.docx]

Supplementary file 1

Details of the studies on the effect of Pegvaliase treatment on mood and neurocognitive functioning

Harding et al. compared adults treated with either 20 mg/kg or 40 mg/kg Pegvaliase with a placebo group in a randomised (2:1 active:placebo) phase 3 study across 8 weeks (46). The study cohort was comprised of 86 adults previously receiving Pegvaliase treatment (in prior phase 2 or phase 3 studies), the randomisation resulted in one third of population discontinuing Pegvaliase for the duration of the 8-week study. Neuropsychological assessments included the inattention subscale (IA) of the ADHD RS-IV (clinician administered), the Profile of Mood States (POMS) score (including PKU-specific POMS score and PKU-POMS confusion subscale score). IA scores range from 0-27, with higher scores corresponding to more inattention symptoms. Cognitive testing was with 3 pre-defined tasks from the Cambridge Neuropsychological Test Automated Battery (CANTAB) considered relevant for PKU (stop signal task reaction time, rapid visual processing mean response latency, and spatial working memory). No deterioration in neuropsychiatric or neurocognitive symptoms was reported in those participants in whom Pegvaliase was withdrawn for 8 weeks (despite a significant increase in blood Phe concentration from < 600 µmol/L to > 1000 µmol/L). CANTAB testing was carried out in 9 participants (6 Pegvaliase: 3 placebo). This showed a difference (increase) in reaction time, that just reached significance, p=0.0497, in the stop signal task in the group of 3 individuals who switched from Pegvaliase to placebo.

Thomas et al. (45) reported outcomes in 261 adults that started treatment with Pegvaliase, 169 of whom completed a long-term extension study and underwent the same neuropsychological testing as above. Results were reported after 12 (178 participants) and 24 (89 participants) months of treatment. The inattention subscale of the ADHD RS-IV score fell from baseline (9.8 ± 6.1) to 12 months (5.0 ± 4.9) and was maintained at 24 months (4.5 ± 4.7). The POMS score at baseline (170 participants) was normal (35.7 ± 30.7) but decreased further to 18.3 ± 29.6 by 24 months (90 participants). No statistical analyses were reported in this paper, but more detailed intention to treat analyses were provided for 156 of these individuals in a paper by Bilder et al. (47). An IA score of ≥10 was chosen to indicate inattention likely to impede daily functioning and the group was analysed as a whole (N=156), as those with IA scores ≤ 9 (N=85), and as those with IA scores ≥ 10 at baseline (N=71), prior to treatment with Pegvaliase. All three groups showed improvement in IA scores with lowering of plasma Phe, which plateaued after about 18 months of Pegvaliase treatment. The improvement was most marked for those in the initial IA subgroup (scores ≥ 10), with a mean change in IA score from 15.3 to 5.1. There was a stepwise reduction in IA scores across quartiles of plasma Phe reduction. Interestingly, this IA score reduction occurred even in quartile four – despite this group having minimal plasma Phe improvement or even an increase in plasma Phe (change from baseline Phe -248 to + 934 µmol/L, in the IA subgroup). Possible reasons given include the Hawthorne effect (study participants reporting improvement as they are aware of being observed), and / or a positive effect of increased dietary protein intake. The smallest intervention study reporting on cognitive functioning before (plasma Phe levels >1300 umol/L) and during treatment with Pegvaliase (plasma Phe levels <360 umol/L) was that of Burlina at all in 3 patients. In all three at least 70% of the scores on the neuropsychological test domains were within the normal range and the shifts were mostly within the normal reference range, without discussing a potential learning effect of repeated testing (48).
